# Dose-Dependent Cytotoxic Effects of Boldine in HepG-2 Cells—Telomerase Inhibition and Apoptosis Induction

**DOI:** 10.3390/molecules20033730

**Published:** 2015-02-24

**Authors:** Sakineh Kazemi Noureini, Michael Wink

**Affiliations:** 1 Deptartment of Biology, Faculty of Basic Sciences, Hakim Sabzevari University, P.O. Box 397, Sabzevar, 9617966376 Iran; 2 Department of Biology, Institute of Pharmacy and Molecular Biotechnology, Heidelberg University, INF 364, 69120, Heidelberg, Germany

**Keywords:** boldine, chemoprevention, telomerase, apoptosis, senescence

## Abstract

Plant metabolites are valuable sources of novel therapeutic compounds. In an anti-telomerase screening study of plant secondary metabolites, the aporphine alkaloid boldine (1,10-dimethoxy-2,9-dihydroxyaporphine) exhibited a dose and time dependent cytotoxicity against hepatocarcinoma HepG-2 cells. Here we focus on the modes and mechanisms of the growth-limiting effects of this compound. Telomerase activity and expression level of some related genes were estimated by real-time PCR. Modes of cell death also were examined by microscopic inspection, staining methods and by evaluating the expression level of some critically relevant genes. The growth inhibition was correlated with down-regulation of the catalytic subunit of telomerase (hTERT) gene (*p* < 0.01) and the corresponding reduction of telomerase activity in sub-cytotoxic concentrations of boldine (*p* < 0.002). However, various modes of cell death were stimulated, depending on the concentration of boldine. Very low concentrations of boldine over a few passages resulted in an accumulation of senescent cells so that HepG-2 cells lost their immortality. Moreover, boldine induced apoptosis concomitantly with increasing the expression of bax/bcl2 (*p* < 0.02) and p21 (*p* < 0.01) genes. Boldine might thus be an interesting candidate as a potential natural compound that suppresses telomerase activity in non-toxic concentrations.

## 1. Introduction

The terminal DNA at chromosome ends, known as telomeres, progressively shorten during each cell division and thus limit the replicative lifespan of dividing cells [1]. It is believed that the continuous proliferation and avoidance of replicative senescence in many cancer cells correlates with their ability to maintain their telomeres by activation of telomerase. This ribonucleoprotein is a special reverse transcriptase that carries out the elongation of telomeres in most immortal cells [2]. Telomerase exhibits extremely low or even undetectable activity in most normal somatic cells, whereas it is strongly reactivated in over 85% of human tumors [3]. Therefore, telomerase has been proposed as a critical anticancer target and several telomerase inhibitors have shown the potential to be used in cancer therapy by disrupting the replicative capacity of cancer cells. Some small synthetic molecules that inhibit telomerase have already been reported [4], often by interacting with the G-quadruplex structure of telomeres [5] or the telomerase ribonucleoprotein [6]. However, we believe that potential telomerase inhibitors and telomere-interfering compounds can be found among plant secondary metabolites, which mainly serve as defensive compounds that protect plant cells against various stresses. Cancer chemoprevention and above all using natural sources seems undoubtedly a promising strategy against cancer.

**Figure 1:**
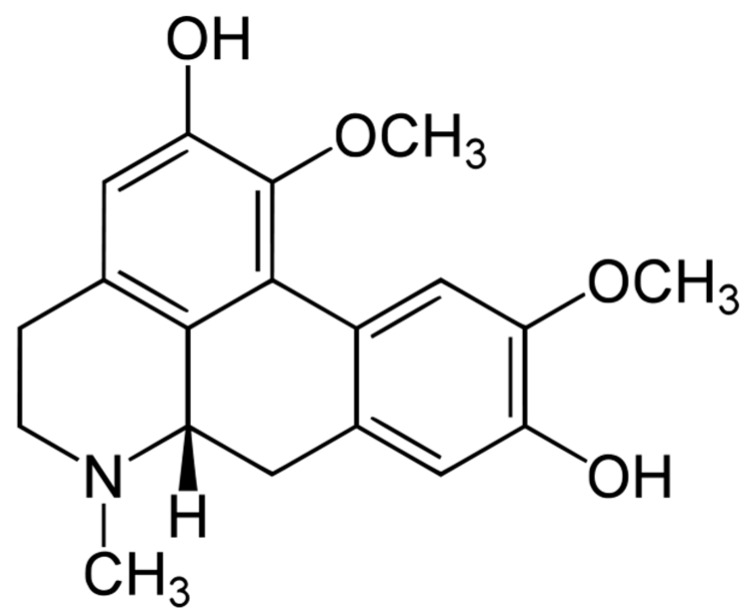
Boldine.

In our preliminary screening of anti-telomerase compounds among plant secondary metabolites [7,8,9], boldine (1,10-dimethoxy-2,9-dihydroxyaporphine, Figure 1), a natural aporphine alkaloid found mainly in the Chilean medicinal plant *Peumus boldo* showed a dose- and time-dependent antiproliferative effect in several cells. In addition to its antioxidative properties, boldine exhibits several other pharmacological activities such as anti-inflammatory, antipyretic, antiatherogenic, antiplatelet, antitumor, cytoprotective [10] and anti-tyrosinase effects [11]. This compound has shown to attenuate brain mitochondrial dysfunction induced by catecholamine oxidation [12]. Aporphine alkaloids in general exhibit a wide range of biological activities such as antiproliferative properties in a number of cancer and non-cancer cell lines [13] and inhibition of topoisomerase I or II [14]. This study was focused on an evaluation of the cytotoxicity and antiproliferative effect of this compound with special reference to telomerase inhibition and induction of apoptosis.

## 2. Results and Discussion

### 2.1. Dose and Time-Dependent Cytotoxicity of Boldine in HepG-2 Cells 

**Figure 2:**
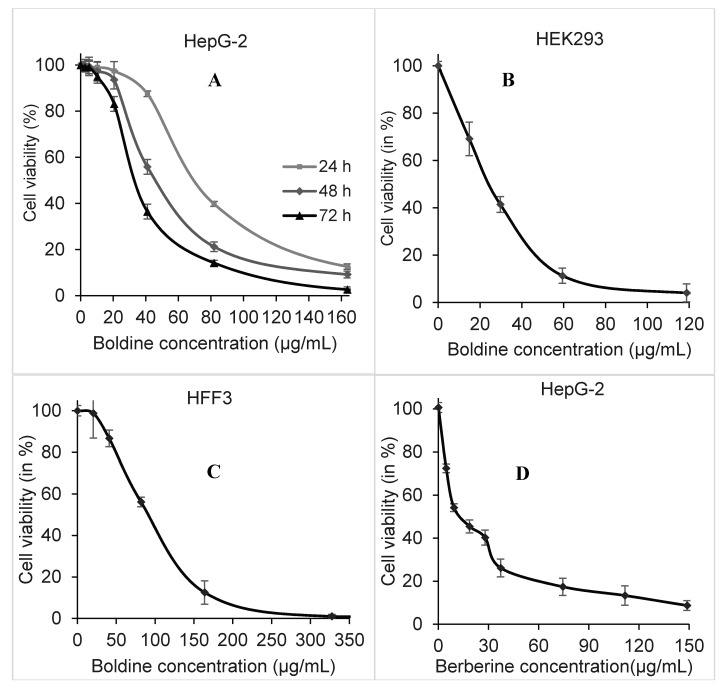
Dose responding viability of HepG-2 cells after 24 (light gray), 48 (gray) and 72 (black) h treatment with boldine (**A**). Cell viability of HEK293 (**B**) and HFF3 cells (**C**) after 48 h treatment with boldine. HepG-2 viability after 48 h treatment with berberine using MTT (**D**).

Cytotoxicity of boldine in the human hepatocellular carcinoma cell line HepG-2, human embryonic kidney HEK293 cells and normal human fibroblast HFF3 cells was investigated using the MTT method. Boldine showed a time- and dose-dependent cytotoxicity in HepG-2 cells. The IC_50_ value of this compound in HepG2 cells after 48 h treatment was estimated at 55.66 ± 1.3 μg/mL, equal to 170 ± 4 µM *p* < 0.001 (Figure 2A). In comparison, after 48 h treatment, the HEK 293 cells were more sensitive (IC_50_ 32.74 ± 2.2 µg/mL *p* < 0.002, Figure 2B) and human fibroblasts (IC_50_ 95 ± 2.5 µg/mL *p* < 0.045, Figure 2C) less sensitive than HepG-2 cells. However, the DNA-intercalating compound berberine was more toxic than boldine, with an IC_50_ of 14.87 ± 1.2 µg/mL *p* < 0.005 in HepG-2 cells after 48 h exposure (Figure 2D).

Boldine showed a moderate time- and dose-dependent inhibition of proliferation. This effect is stronger in immortal cancer cells than in human foreskin fibroblasts, while embryonic kidney cells are more sensitive than cancer cells. Both HepG-2 and HEK293 have an active telomerase, while HFF3 shows no detectable telomerase activity; therefore, we decided to explore whether telomerase was affected. 

### 2.2. Boldine Effectively Suppresses Telomerase Mainly by hTERT Down-Regulation

**Figure 3:**
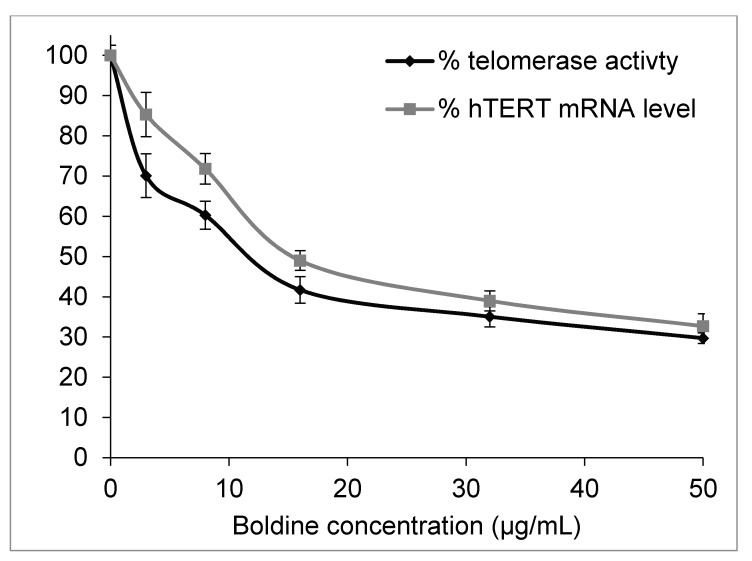
Dose-dependent inhibition of telomerase activity and hTERT mRNA levels in HepG-2 cells 48 h treated with boldine (*P* values are < 0.002 and 0.01 respectively).

A real-time quantitative telomeric repeat amplification protocol (q-TRAP assay) was employed to quantify telomerase activity in HepG-2 cells. A 48 h treatment of the cells with boldine significantly decreased telomerase activity so that the enzyme activity decreased to 50% as compared to untreated cells (*p* < 0.002) when treated with boldine concentration of 11.4 ± 1.5 µg/mL (equal to 34.8 ± 3.4 µM) (Figure 3). 

Telomerase is mainly regulated at the transcription level of the hTERT gene that encodes the catalytic subunit [15]. Real-time PCR experiments presented in Figure 3 show an obvious dose-dependent reduction of hTERT expression in HepG-2 cells under boldine treatment (*p* < 0.01). Therefore, results from q-TRAP and qRT-PCR methods showed that the telomerase inhibition by boldine correlates with a down-regulation of the hTERT gene. However, telomerase activity reduction was slightly greater than would be expected based on the hTERT mRNA level. The difference is likely due to a direct interaction of boldine with the protein, or an involvement of some other aspects of telomerase regulation including alternative splicing.

As seen in Figure 2A boldine at this concentration has almost no cytotoxicity in HepG-2 cells. It is noteworthy that most of the synthetic anti-telomerase compounds are very toxic (with IC_50_ values in the nM to µM range) [4,16], so it is important to find new compounds that inhibit telomerase at non-toxic concentrations. The same method estimated a reduction of telomerase activity to 50% by 5.57 ± 0.6 µg/mL *p* < 0.01 berberine [17], at which cell viability of HepG-2 is reduced to 25%–30% in comparison with untreated cells as seen in Figure 1D. Berberine has been used as a known telomerase inhibitor [18], however it is a very potent DNA intercalating agent with a very high affinity so that it has been employed for DNA staining [19]. A higher activity of telomerase and long telomeres in advanced hepatocellular carcinoma has been reported to be associated with poor prognosis and invasive capacity [20,21]. Inhibition of telomerase by boldine in HepG-2 cells might enable telomere shrinkage and limit cell invasion.

### 2.3. Boldine Induces Apoptosis in HepG-2 Cells

**Figure 4:**
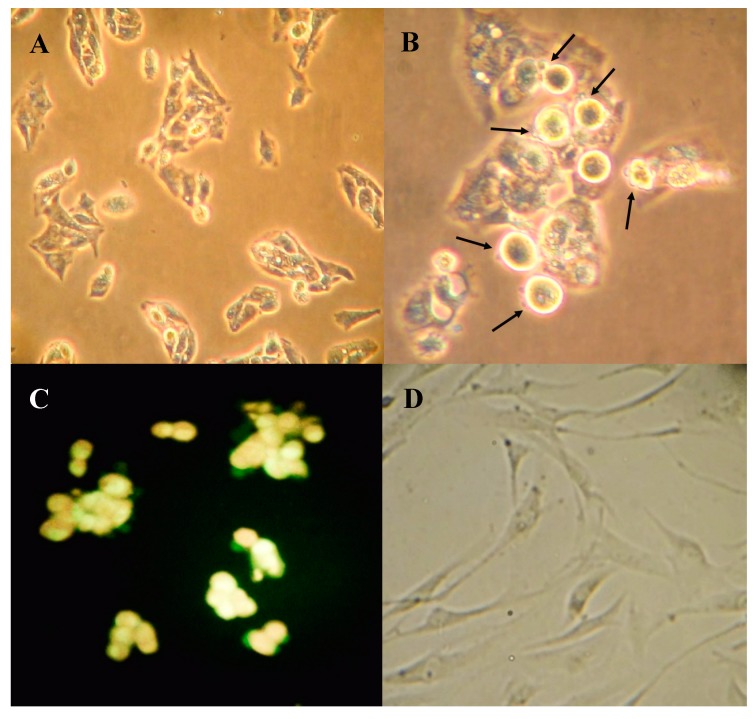

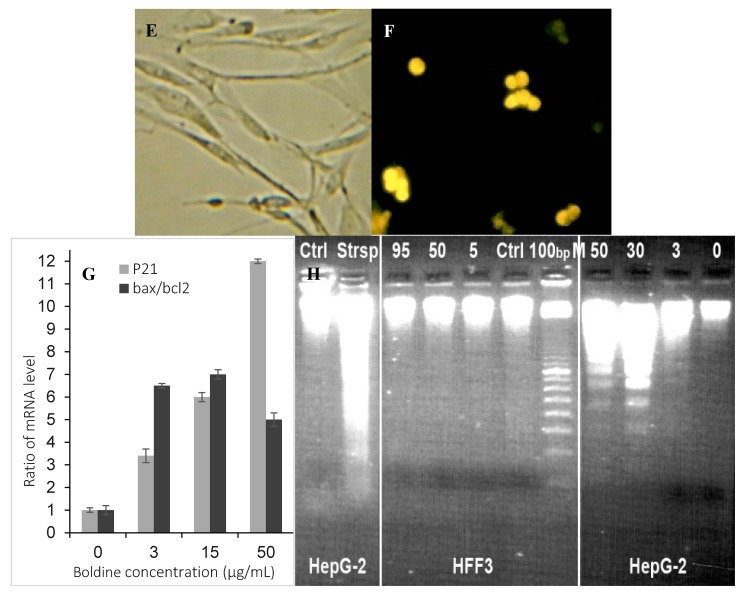
HepG-2 and HFF cells seeded on cover slip (**A** and **D** respectively) and treated with 50 µg/mL boldine for 48 h (**B** and **E** respectively), fixed and viewed with inverted phase microscope. Fluorescence microscopy after staining with acridine orange and ethidium bromide (**C** and **F** respectively). The magnification is 200× in A and D, E, F and 400× in B and C. Relative expression of p21 *p* < 0.01, and bax/bxl2 in HepG-2 cells treated with boldine for 48 h; *p* < 0.02 (**G**). Apoptotic DNA fragmentation in HepG-2 cells after boldine treatment (from right to left: 0 (untreated control), 3, 30, 50 µg/mL, respectively) separated in agarose gel electrophoresis. HFF3 treated with 0, 5, 50 and 95 µg/mL showed no DNA fragmentation. DNA sample from HepG2 cells treated with staurosporin as a known apoptosis inducing compound and untreated cells, respectively (**H**).

Morphological changes were apparent in HepG-2 cells after 48 h treatment with 50 µg/mL boldine as viewed with phase contrast microscope (Figure 4B) in comparison with untreated HepG-2 cells (Figure 4A). At this concentration, boldine effectively induced apoptosis in HepG-2 cells. Staining with acridine orange/ethidium bromide also showed membrane blebbing and nuclear condensation. However, apoptosis was stimulated in HepG-2 cells only at high concentrations (Figure 4C). HFF3 as a normal cell model (Figure 4D) was also used to check the toxicity and apoptogenic effect of boldine. No considerable sign of apoptosis was seen in HFF3 cells after 48 h treatment with 95 µg/mL boldine (Figure 4E,F). Boldine significantly increased bax/bcl2 mRNA ratio up to several fold, *p* < 0.02 in HepG-2 cells (Figure 4G). A typical DNA fragmentation pattern in treated HepG-2 cells confirms apoptosis induction by boldine (Figure 4H). Both of these effects are substantial at high concentrations of boldine. There are several other natural/synthetic compounds with both apoptosis induction and telomerase inhibition in different cancer cells [22,23,24,25,26,27]. However, boldine is a strong anti-oxidant and supposed to prevent ultraviolet mediated DNA damage [28,29]. Therefore, apoptosis induction by boldine that was observed only at high concentrations is probably explained with its pro-oxidant properties at high doses [30,31].

On the other hand, telomerase is believed to play critical roles in the regulation of apoptosis in a telomere-independent manner [32], probably by targeting mitochondria [33]. It is also known that down-regulation of hTERT can sensitize mitochondrial apoptosis pathways, not by telomere shortening but rather by activation of Bax [34,35]. We suggest that boldine, which at high concentrations can cause pro-oxidative effects [31], may trigger nuclear export of hTERT and stimulate apoptosis.

### 2.4. Boldine Induces p21 in HepG-2 Cells 

p21 (also known as p21WAF1/Cip1) is one of the most relevant genes that affect cell senescence, hTERT regulation and apoptosis. The relative expression level of this gene was assessed in boldine-treated cells. Real-time RT-PCR analysis showed a significantly dose-dependent increase of p21 gene in HepG-2 cells when treated with boldine, *p* < 0.01 (Figure 4G).

p21 induction results in apoptosis stimulation probably by overexpression of bax [36], both of which were observed in the boldine treated cells in this study. It is known that cell division is strongly suppressed by p21 through interaction with proliferating cell nuclear antigen (PCNA) [37] and repression of cyclin-dependent kinase 1 (CDK1) or cell division cycle protein 2 homolog (CDC2), checkpoint kinase 1 (CHEK1) and TERT [38]. Our data may explain down-regulation of hTERT transcription as due to inhibition of its promoter through p21 induction in the treated cells. *Vice versa*, down-regulation of hTERT expression can result in an elevated p53 and p21 transcription and a decrease in cellular proliferation [39]. In MCF-7 cells, the down-regulation of telomerase by the alkaloid harmine induced an overexpression of p53/p21 pathway resulting in an accelerated senescence phenotype of the cancer cells [40], similar to the results seen in our boldine experiments. 

### 2.5. Low Non-Toxic Concentration of Boldine Accelerates Senescence in HepG-2 Cells

**Figure 5:**
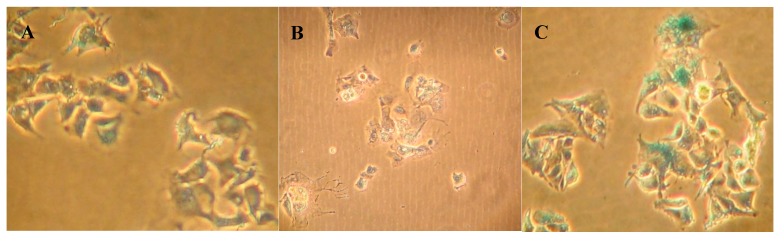

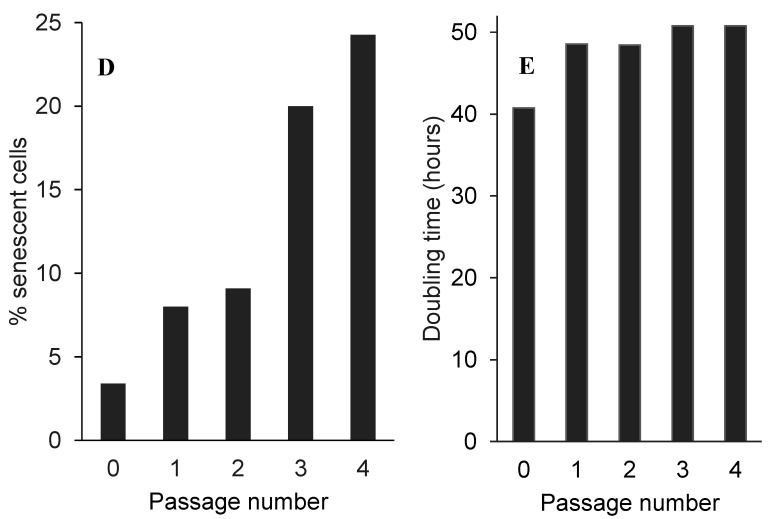
Morphology of un-treated HepG-2 cells after β-galactosidase staining (**A**). Treated HepG-2 cells after four treatments with 3 µg/mL boldine 48 h in each of the consecutive passage (**B**) and after β-galactosidase staining (**C**). The magnification is 200× in all pictures. Percent of positively stained HepG-2 cells for β-galactosidase activity (**D**) and HepG2 cell doubling time in long-term treating with 3 µg/mL boldine 48 h per passage as described in text (**E**); each bar shows the average of data collected from two independent experiments each in duplicates.

A repeated exposure of HepG-2 cells to a low non-toxic concentration of boldine (3 µg/mL) 48 h per passage, resulted in a longer cell doubling times and clear morphological changes. Treated cells exhibited very large cytoplasm-to-nucleus ratios relative to untreated cells (Figure 5A,B). The treated cells stained dark blue because of the β-galactosidase activity detected by using the Dimri staining method [41] are considered as senescent cells (Figure 5C). In two logical repeats of the experiment each in duplicates the percentage of these senescent cells after four consecutive treatments increased to 28% ± 2.1% in comparison with 3.5% ± 0.5% in untreated cells (Figure 5D). The doubling time increased to 50 ± 1.2 h in the treated cells in comparison with 41 ± 0.4 h in control cells (Figure 5E). 

Our β-galactosidase staining and quantitative real-time PCR results suggest alteration of gene expression pattern towards induction of senescence under treatment with boldine. It is known that several pathways including senescence may change by induction of p21 [42,43]. Therefore, it can be concluded that the elevated p21 mRNA level by boldine may stimulate cell cycle arrest and accelerate senescence. 

## 3. Experimental Section 

### 3.1. Cell Culture

Human hepatocarcinoma cell line (HepG-2), human embryonic kidney 293 cell line (HEK293) came from DSMZ (German Collection of Microorganisms and Cell Cultures, Leibniz, Germany) and normal human foreskin fibroblast cells (HFF3), kindly provided by the Royan Institute (Tehran, Iran). HepG-2 cells established by ATCC are adherent, epithelial-like cells growing as monolayers widely used as a well-known model for hepatocarcinoma in anti-cancer research projects. HepG-2 and HEK293 are telomerase-positive cell lines, while fibroblast cells have no detectable telomerase activity. All cell lines were cultivated in Dulbecco’s modified Eagle’s medium (DMEM high glucose with stable glutamine) supplemented with 10% fetal bovine serum (FBS gold), 100 U/mL penicillin, and 100 μg/mL streptomycin (all the mentioned materials came from PAA Company, Pasching, Austria) in a humidified atmosphere containing 5% CO_2_ at 37° C. All cell lines were subcultured routinely when they reached almost 80% confluence. The cytotoxicity experiments were performed with cells in the logarithmic growth phase. 

To study long-term effects of boldine on growth of HepG2 cells, treated or untreated cells were seeded into 75 cm^2^ tissue culture flask at 1 × 10^6^ for 4–5 d until 70%–80% confluence, then trypsinized and counted. Each time 1 × 10^6^ cells were re-plated into new culture flasks but in each passage, the treated series were incubated at the desired concentration of boldine for 48 h and afterward cells fed with fresh normal medium. A small part of the harvested cells in each passage were plated for β-galactosidase assay. The doubling time was calculated by DT=log_10_2T/(log_10_n_f_ − log_10_n_i_), where n_i_ is the initial number of cells and n_f_ is the final number of cells at each passage and T is time in h. This experiment was repeated two times, each in duplicates.

Berberine and boldine (purum ≥ 98%) were purchased from Sigma-Aldrich (Munich, Germany) and Fluka (Seelze, Germany), respectively, and dissolved in absolute ethanol (Merck, Darmstadt, Germany) at a concentration of 50 mM (stock solution) and stored at −20 °C until use. Each stock solution was serially diluted in medium before use so that the maximum final concentration of ethanol in cell cultures did not exceed 0.1%. Berberine was used as a known compound with telomerase inhibitory effect [18].

### 3.2. Cytotoxicity Assay

Cell viability was evaluated using the MTT (3-(4,5-dimethylthiazol-2-yl)-2,5-diphenyltetrazolium bromide, Sigma-Aldrich) assay [44]. Briefly, cells were seeded in 96-well plates at a density of 1 × 10^4^ cells/well and treated with different concentrations of the desired compound. After various incubation times, MTT (0.5 mg/mL final concentration) was added and incubated 4 h to be reduced to blue formazan by viable cells. The concentration of formazan after dissolving in isopropanol was determined by measuring its absorbance at 570 nm using a plate reader (BioTek, Winooski, VT, USA). Data were analyzed using Gen5 software version 1.06 (BioTek, Winooski, VT, USA). The assays were carried out at least three times, and each included three samples per point. The concentration of compound required to inhibit cell growth to 50% of untreated controls, defined as the IC_50_ value, was determined from the dose-response curves. 

In subsequent experiments HepG-2 cells were exposed to three different concentrations; a relatively low concentration correlated with low cytotoxicity of boldine in MTT test, a second concentration was equal to IC50 to have the logical maximum treatment, and a third concentration around the midpoint of the cytotoxicity curve. Treatment duration time was set to 48 h to allow adequate time for cycling cells, because telomerase exists in a very low copy number and is active only in a short period during S phase.

### 3.3. Telomerase Assay (SYBR Green q-TRAP Assay)

Telomerase activity was determined based on a SYBR-Green quantitative-telomeric repeat amplification protocol (q-TRAP) method as described earlier [7]. Briefly, the cancer cell lines after 48 h incubation with various concentrations of boldine and/or berberine were washed with phosphate buffer saline (PBS), collected and lysed as described earlier. Protein concentrations were estimated with a microBradford assay using a plate reader (BioTek). Using SYBR Green PCR Master Mix (GenetBio, Gobiz, South Korea), q-TRAP assay was performed in a real-time thermal cycler Rotor Gene 3000 (Corbett Research, Sydney, Australia) as previously reported. The threshold cycle values (Ct) for each sample were determined from amplification plots (log increase in fluorescence *versus* cycle number) as analyzed with Rotor Gene 6.01 (Qiagen) and compared with standard curves generated from the serially diluted cell lysates of untreated control samples. This experiment was repeated three times independently and each time contained at least 3 samples per point.

### 3.4. Total RNA Isolation, cDNA Synthesis, and Real-Time PCR

Total RNA was isolated from control and 48 h treated cells using RNX-Plus solution (SinaClone BioScience, Tehran, Iran) according to the manufacturer’s instructions. Reverse transcription of 2 μg of total RNA to cDNA was carried out using MMULV reverse transcriptase (Vivantis, Subang Jaya, Malaysia). The expression level of hTERT, bax, bcl2, p21 and β2-microglobulin genes (as a house keeping control gene) was detected using the quantitative reverse transcription polymerase chain reaction (qRT-PCR) method as published earlier [7]. Briefly, one µL of each cDNA sample was subjected to PCR-amplification reaction including SYBR Green PCR Master Mix (2×) and 5 pmol of each exon-junction spanning primer pairs. The mRNA copy number of each gene was normalized to that of the housekeeping gene, β2-microglobulin, using the related standard curves from Rotor Gene 6.01 software and then compared with that of untreated controls. This experiment was repeated three times independently, each contained at least three replicates for every point.

### 3.5. DNA ladder Formation

The cells were treated with the specified concentrations of boldine for 48 h. Cellular DNA was isolated and subjected to agarose gel electrophoresis followed by visualization of bands by ethidium bromide staining [45]. The data shown here is a representative result from three independent experiments with almost similar pattern.

### 3.6. Fluorescence Microscopy of Apoptotic Cells

Morphological assessment of apoptotic cells was performed using an acridine orange/ethidium bromide double staining method [46] with some modifications. HepG-2 cells were seeded on cover slips and then incubated with 0 and 50 µg/mL boldine for 48 h. After incubation, cells were washed with PBS, fixed and stained with acridine orange/ethidium bromide solution (1/1 v/v) at a final concentration of 10 µg/mL. The nuclear morphology of the cells was observed with a fluorescence microscope (Olympus, Tokyo, Japan). 

### 3.7. β-Galactosidase Staining as a Senescence Marker

Control and treated cells were fixed for 3–5 min at room temperature in 2% formaldehyde/0.2% glutaraldehyde, washed twice with PBS and incubated at 37 °C (without CO_2_) with fresh senescence-associated β-gal (SA-β-gal) stain solution as described by Dimri *et al.* [41]. Staining was achieved in 2–4 h and positively stained cells (dark blue) were counted in at least five microscopic fields including almost 500 cells. The ratio of the stained cells to the total number of cells was expressed as percentage of senescent cells. 

### 3.8. Statistical Analysis 

Statistical analysis was performed by using an ANOVA test and *p* < 0.05 was considered significant. Data were presented as mean ± SEM from at least three independent experiments.

## 4. Conclusions 

In conclusion, our data demonstrate the beneficial antiproliferative properties of boldine, which suppresses immortality of Hep-G2 cells at non-toxic concentrations most likely through inhibition of telomerase and induction of senescence. At high concentrations, boldine induces apoptosis. These effects are supported by a recent study that reported the induction of cell cycle arrest and apoptosis in T24 human bladder cancer cells by boldine via regulation of ERK, AKT, and GSK-3β [47]. Another study has shown that boldine at a dose of 100 mg /kg body weight in an animal model of breast cancer is well-tolerated and intraperitoneal injection of boldine (50 or 100 mg/kg) significantly reduces tumor size [48]. The authors have described G2/M arrest, activation of caspase-9 and caspase-3/7 (but not caspase-8) in the metastatic breast cancer cell line MDA-MB-231 by boldine, which also down-regulates Bcl-2 and heat shock protein 70 and up-regulates Bax. This study strongly suggests that boldine, a potent and hardly toxic antioxidant, could serve as a valuable anti-cancer agent. Modifications of the skeletal structure of boldine could lead to enhanced potency against cancerous cells. Potential modifications of the molecule can probably provide a more selective telomerase inhibitors. 
